# The Concealing Effect of COVID-19: Unveiling the Surge in Community-Acquired Infections and Its Association With Diabetic Ketosis During the Pandemic

**DOI:** 10.7759/cureus.60967

**Published:** 2024-05-24

**Authors:** Ach Taieb, Jihen Bouzayene, Sawsen Nouira, Nadia Ben Lasfar, Amel Amara, Asma Gorchane, Wissem Hachfi, Koussay Ach

**Affiliations:** 1 Endocrinology, Farhat Hached University Hospital, Sousse, TUN; 2 Laboratory of Exercise Physiology and Pathophysiology, Farhat Hached University Hospital, Sousse, TUN; 3 Family Medicine, Farhat Hached University Hospital, Sousse, TUN; 4 Infectious Diseases, Farhat Hached University Hospital, Sousse, TUN; 5 Family and Community Medicine, Faculty of Medicine of Sousse, University of Sousse, Sousse, TUN; 6 Endocrinology and Diabetes, Farhat Hached University Hospital, Sousse, TUN

**Keywords:** sars-cov-2, ketosis, diabetes, infections, covid-19

## Abstract

Background

Some studies suggest that the SARS-CoV-2 pandemic has contributed to diverting attention from other community-acquired infections (CAIs), leading to an increase in their incidence and severity. Our study aimed to describe and compare clinical features of CAI before and during the pandemic as a factor precipitating diabetes ketoacidosis (DKA).

Methodology

We included 250 patients who presented with DKA due to CAIs, other than SARS-CoV-2, divided into two distinct groups: 100 patients (G1) who consulted two years before the pandemic, and 150 patients (G2) who consulted during the SARS-CoV-2 pandemic. Cases in both groups were matched for age and type and duration of diabetes. Primary outcomes were a longer diagnosis delay and more severe DKA in G2 during the pandemic. Secondary outcomes included blood test results, duration of ketosis, duration of antibiotic therapy, and diabetes treatment.

Results

The diagnosis and treatment delays were longer for patients seeking medical care during the pandemic (p < 0.001). The duration of DKA was also significantly longer in the G2 group (p = 0.007). During the pandemic, patients’ blood tests showed more anomalies with higher glycated hemoglobin (p = 0.02), C-reactive protein (p = 0.001), and lymphocytosis (p = 0.016). The duration of antibiotic therapy was also significantly longer in G2 (p = 0.01).

Conclusions

This study showed the impact of the COVID-19 pandemic on the management of diseases other than SARS-CoV-2. Indeed, several factors played a part in the increased incidence of CAIs, which were more severe than in the pre-pandemic period. These included fear of contagion, confinement, and physicians’ preoccupation with the pandemic.

## Introduction

Diabetes mellitus (DM) is a chronic disease characterized by hyperglycemia resulting from insufficient insulin action and/or secretion [1]. The prevalence of DM is rising rapidly worldwide, placing a high economic burden on patients and society in general [1]. More than 425 million people worldwide suffer from this disease, and projections show that this figure will rise to 629 million by 2045 [2,3]. Over time, hyperglycemia can damage both large and small blood vessels, leading to an increased risk of microvascular and macrovascular complications [3]. In addition to the comorbidities associated with micro- and macro-angiopathies, DM is commonly associated with infections. The main mechanism is the immunosuppression resulting from chronic hyperglycemia, which alters the immune cell response. Several sites of infection have been described in association with DM, including skin, gastrointestinal, urinary, and respiratory infections, with significantly increased hospitalization and mortality rates [1]. Infections in diabetic patients have long been considered one of the causes of increased morbidity and mortality. They are a well-known factor in glycemic imbalance and ketosis [1]. Taieb et al. found that viral infection was the main precipitating factor for inaugural diabetic ketoacidosis (DKA) in diabetes [4]. In addition to the life-threatening consequences of SARS-CoV-2 pneumonia, the pandemic also had an indirect impact on the monitoring of these patients’ chronic pathologies. As a result, many patients had to reschedule a consultation appointment, following health instructions to avoid contagion. This disrupted the glycemic balance of diabetic patients, increasing their state of immunodepression and the risk of further community-acquired infections (CAIs) [5]. However, during the pandemic, the attention of all healthcare workers was diverted to the management of SARS-CoV-2, leading to diagnostic problems and delays in the management of CAI. There are no concrete data on the increased risk of CAI other than SARS-CoV-2 in our country and on the African continent.

This study aims to compare the prevalence of CAIs as a factor of DKA before and during the pandemic, analyze and compare the severity of DKA between these two periods, and identify the most frequent site of CAI occurrence before and during the SARS-CoV-2 pandemic.

## Materials and methods

This matched cohort study was conducted at the Department of Endocrinology of the Farhat Hached University Hospital in Sousse, Tunisia, as a single-center study. It is a university hospital that provides healthcare for the entire central region of Tunisia. We analyzed data from March 2018 to March 2020, and two years during the SARS-CoV-2 pandemic, from March 2020 to March 2022.

We included 250 patients with DKA due to CAIs, other than SARS-CoV-2 infection. We divided the study population into two distinct groups, according to the period of infection diagnosis: Group 1 (G1): from March 01, 2018, to March 31, 2020, and Group 2 (G2): from April 01, 2020, to March 31, 2022 (the SARS-CoV-2 pandemic period). All patients included in the study during the pandemic tested negative for COVID-19. Diagnostic methods were either virological tests (real-time reverse transcription polymerase chain reaction) or rapid diagnostic tests. Cases were matched in pairwise fashion according to age and type and duration of diabetes.

All patients with unbalanced diabetes of non-infectious causes, pregnant patients, and patients under 18 years of age were not included. We excluded all patients with a history of organ transplantation, patients on immunosuppressants, patients on long-term antibiotic and corticosteroid therapy, or patients with insufficient data in their medical files.

Cases were selected by conducting a search in the electronic admissions system of the Department of Endocrinology during the selected periods. Data were collected using an information chart including demographics (age and gender, family and personal medical history, assessment of DM (type, duration, and control) and its complications, and the infectious episode with its clinical and biological features.

Continuous variables were tested for normal distribution before analysis. Clinical and biological parameters were expressed as mean ± SD. To compare patients in the two subgroups, we used Student’s t-test for quantitative variables and the chi-square test, when validity conditions allowed, for qualitative variables. Missing data points were removed from the final analyses. The significance level (p) was set at 5%. All data were analyzed using SPSS software version 23.0 (IBM Corp., Armonk, NY, USA).

## Results

As we have already described, DKA cases triggered by CAIs other than SARS-CoV-2 were more frequent during the pandemic, 100 for G1 and 150 for G2. The majority of patients in G1 (94%; N = 94) were diagnosed on time compared to 56% (N = 84) in G2, revealing a significant delay in diagnosis (p < 0.001) during the pandemic.

The majority of patients (87%; N = 217) were type 2 diabetics in both groups. Diabetes was newly diagnosed in 20% (N = 20) of cases in G1 and 22% (N = 33) of cases in G2. The mean age of the entire population was 56.32 ± 14.21 years, with a sex ratio (male/female) of 1.11 in the two groups. Personal history of cardiovascular disease was more prevalent among G2 patients (Table 1).

**Table 1:** Sociodemographic characteristics, history, and characteristics of diabetes. G1 = Group 1; G2 = Group 2

Clinical characteristics	G1 (N = 100)	G2 (N = 150)	P-value
Age, mean ± SD	57 ± 15	55.24 ± 13	0.141
Sex ratio	0.85	0.92	
Overweight patients, n (%)	78 (78)	114 (76)	0.09
Inaugural diabetes, n (%)	20 (20)	33 (22)	0.06

Skin and respiratory infections other than SARS-CoV-2 were the most common diagnoses for patients in both study groups.

DKA was more severe in G2, as demonstrated by the comparison of clinical and biological data between the groups. We found that pH was lower in G2 patients at the time of diagnosis (p = 0.0001) and HCO_3^-^_ levels were also lower (p = 0.005).

Mean blood glucose levels on admission were significantly higher in G2 than in G1, with averages of 13.95 ± 5.73 mmol/L for G1 versus 15.25 ± 5.94 mmol/L for G2 (p = 0.03). Mean glycated hemoglobin (HbA1c) was significantly different with an estimated mean value of 8.1% (6.6-9.6) in G1 versus 9.3% (0.7-17.8) in G2 (p = 0.02). The mean C-reactive protein (CRP) level was significantly higher in G2 (115.44) than in G1 (38.02) (p = 0.001) (Table 2).

**Table 2:** Results of biological tests. CRP = C-reactive protein; G1 = Group 1; G2 = Group 2; SD = standard deviation

Biological tests	G1, mean ± SD	G2, mean ± SD	P-value
Lymphocytes (/mm^3^)	1,492.7 ± 551.5	1,673.7 ± 592.8	0.016
pH	7.3 ± 0.1	7.2 ± 0.1	0.0001
HCO_3_^-^ (mmol/L)	15.7 ± 2.6	14.5 ± 3.6	0.005
Glycated hemoglobin	8.1 ± 8.5	9.3 ± 8.5	0.02
CRP	38.02 ± 42.1	115.5 ± 34	0.001

Ketosis duration was significantly different between the two periods (p = 0.007). It was longer for G2, with a mean of 2.5 days versus 2.2 days (Table 3).

**Table 3:** Clinical features of the infection. G1 = Group 1; G2 = Group 2; SD = standard deviation

Clinical features	G1, mean ± SD	G2, mean ± SD	P-value
Duration of emergency room stay (days)	1.95 ± 0.99	2.88 ± 1.53	0.04
Duration of antibiotic treatment (days)	9.8 ± 3	14 ± 4	0.01
Duration of ketosis (days)	2.2 ± 1.5	2.5 ± 1.6	0.007

The average duration of antibiotics prescribed for the CAI was significantly longer in the pandemic period, at 9.8 ± 3 days for G1 versus 14 ± 4 days for G2 (p = 0.01). The most commonly used antibiotic for G1 and G2 was cefotaxime, estimated at 25% (N = 25) for G1 versus 19.3% (N = 28) for G2.

The majority of patients (98.8%; N = 247) changed their diabetes treatment upon hospital discharge. The two groups were comparable in terms of the treatments prescribed (p = 0.07).

## Discussion

Despite recent advancements in diabetes and infectious disease management, patients with DM still have a higher risk of CAIs. Specifically, poorly controlled diabetes increases the risk of serious skin, bone, eye, ear, gastrointestinal, urinary, and respiratory infections, among others, with significantly higher rates of hospitalization and death [6].

A study by Seshasai et al. examined the risk of death related to CAIs in over 800,000 participants and found that infectious diseases significantly reduced the life expectancy of patients with diabetes [7]. More recent epidemiological studies have explored the relationship between poor diabetes control and CAIs using a British primary care database of over 85,000 diabetic patients [6,8]. Their results suggest that uncontrolled diabetes is strongly associated with serious infections. Infectious diseases and DM have a bidirectional relationship. On one hand, poor glycemic control increases the risk of infections, and, on the other, infectious diseases are sometimes the trigger for metabolic decompensation up to and including DKA in both newly diagnosed and long-standing patients [9,10]. Hyperglycemia reduces immune system activity and is responsible for changes in tissue, skin, and blood circulation, which increases the risk of CAIs [11]. If left untreated, chronic hyperglycemia impairs leukocyte function and increases the virulence of infections.

Although there are distinct mechanisms to explain the increased incidence of infectious diseases in diabetic patients, the common pathogenic mechanisms are linked to immune dysfunction. Although humoral immunity does not appear to be significantly impaired in people with DM, a large number of studies suggest changes in cell-mediated immunity, such as chemotactic action, phagocytosis, and cytokine secretion, in both type 1 and type 2 DM [12,13]. For example, significantly lower levels of chemotaxis were observed in leukocytes from type 1 or type 2 diabetics compared with controls [14,15].

The results of our study showed a delay in consultation and diagnosis of the CAI for G2 during the viral pandemic. This delay is attributable to patient inertia and fear of consulting a doctor during the pandemic to avoid contagion by the virus. It could also have been increased by the implementation of confinement during prolonged periods.

In our study, prolonged diagnostic delay of the CAI may explain the severity of DKA during the SARS-CoV-2 pandemic [16]. In a Serbian study by Vorgucin et al., telemedicine, reduced access to healthcare, and confusing DKA symptoms with the digestive form of SARS-CoV-2 were identified as possible reasons for the delayed diagnosis of DKA and its severity [17].

In our study, DKA was more severe during the pandemic, as evidenced by lower pH and bicarbonate levels in G2. The duration of ketosis was also significantly longer in G2. Our results are consistent with those of an American study conducted by Jafari et al. in 2022 [18].

However, the results published by Khan et al. in 2022 do not agree with ours. They noted that biomarkers of DKA severity (blood glucose, pH, bicarbonate, and beta-hydroxybutyric acid levels, as well as ketonuria) were not significantly different between pre-pandemic and pandemic DKA cases [19].

In our study, among biological markers, a significant difference, between the two groups, was observed for lymphocyte, HbA1c, and CRP levels. This may be explained by the delay in diagnosis and management of both the CAI and DM. According to the study conducted by Khan et al., in which several other markers were compared, a significant difference was noted for serum urea, creatinine, hemoglobin, and hematocrit [19].

In our study, we noted an increase in the duration of antibiotic therapy. This is also explained by the delay of infectious management, worsened by the immunosuppression caused by DM.

## Conclusions

The emergence of SARS-CoV-2 occurred in the context of an already existing diabetes pandemic, creating a multitude of challenges for the healthcare system worldwide and adding to the burden of an already overburdened healthcare system. This study illustrates the indirect impact of the pandemic, limiting access to primary healthcare, as many people missed routine examinations and medical appointments. Finally, the emergence of DKA triggered by CAIs during the SARS-CoV-2 pandemic underlines the importance of a comprehensive approach to healthcare that recognizes the complex interplay between infectious and chronic diseases. By adopting an integrated approach, we can develop effective prevention, diagnosis, and management strategies, reducing the burden of the disease and improving the health and well-being of populations worldwide.
